# PET/MRI Imaging in High-Risk Sarcoma: First Findings and Solving Clinical Problems

**DOI:** 10.1155/2013/793927

**Published:** 2013-07-01

**Authors:** Markus K. Schuler, Stephan Richter, Bettina Beuthien-Baumann, Ivan Platzek, Jörg Kotzerke, Jörg van den Hoff, Gerhard Ehninger, Peter Reichardt

**Affiliations:** ^1^Department of Internal Medicine I, University Hospital Carl Gustav Carus, Technical University at Dresden, Fetscherstraße 74, 01307 Dresden, Germany; ^2^Department of Nuclear Medicine, University Hospital Carl Gustav Carus, Technical University at Dresden, 01307 Dresden, Germany; ^3^Department of Radiology, University Hospital Carl Gustav Carus, Technical University at Dresden, 01307 Dresden, Germany; ^4^PET Center, Institute of Radiopharmacy, Helmholtz-Zentrum Dresden-Rossendorf, 01328 Dresden, Germany; ^5^Interdisciplinary Oncology, HELIOS Klinikum Berlin-Buch, 13125 Berlin, Germany

## Abstract

Simultaneous positron emission tomography (PET) and magnetic resonance imaging (MRI) is a new whole-body hybrid PET/MR imaging technique that combines metabolic and cross-sectional diagnostic imaging. Since the use of MRI in imaging of soft-tissue sarcoma is extremely beneficial, investigation of the combined PET/MRI is of great interest. In this paper, we present three cases and first data. Combined PET/MRI technique can support the process of clinical decision-making and give answers to some meaningful questions when treating patients with STS. Therefore, the combined modality of simultaneous PET/MRI offers new pieces to the puzzle of sarcoma treatment.

## 1. Introduction

Simultaneous positron emission tomography (PET) and magnetic resonance imaging (MRI) is a new whole-body hybrid PET/MR imaging technique that combines metabolic and cross-sectional diagnostic imaging. Recently, Buchbender and colleagues [1] presented a review of the literature in the use of PET/MRI in oncology. They also stated that in certain domains like soft-tissue sarcoma (STS) no published reports are yet available.

To further address the potential benefit of combined PET/MRI in a disease, where the soft-tissue contrast in MRI could be extremely beneficial, we want to present our first data and discuss further fields of research.

FDG-PET and PET/CT have been widely investigated in staging and restaging of sarcoma [2, 3]. PET/CT has been investigated in STS for biopsy guidance [4, 5], response assessment [6, 7], and grading [8]. Moreover, whole-body imaging can contribute to the multimodality in treatment of sarcoma [9] and prognosis of patients with STS [10]. PET/CT can also be used to accurately identify local disease recurrence in sarcoma patients [11]. FDG-PET has been demonstrated to be highly superior in detecting bone and lymph node metastases in paediatric sarcoma patients compared to conventional imaging [12], leading to an upstaging in a high percentage of patients [13].

Previously it has been demonstrated that FDG-PET and FDG-PET/CT can be used to predict metastases-free survival in patients with sarcoma of the extremities treated with neoadjuvant chemotherapy (CTX) [7, 14]. PET/CT was also superior to changes in size alone in predicting response to CTX [15]. Yet, there were also findings that do not support the use of PET in metastatic disease [16] and question the benefit of combined PET/CT [17]. However, very promising results were presented by Herrmann et al., showing that PET/CT can also be used as a prognostic marker in STS [18]. For that reason it is obvious to investigate the addition of functional PET to MRI.

MRI is the widely recommended and most valuable imaging technique for soft tissue sarcoma. It preferably gives additional information compared to a CT scan. MR imaging allows superior contrast imaging which can especially contribute to the preoperative situation. It also appears superior in delineating vascular and neural structures from tumour tissue. MRI has also been superior to CT in certain sarcoma subtypes with unique metastatic patterns. Schwab et al. strongly supported the use of MRI to more accurately detect spinal metastases in myxoid liposarcoma [19]. Moreover, MRI does not have any radiation exposure. 

## 2. Results

We present first findings using combined PET/MRI technique to support the process of clinical decision-making. In this brief report, by presenting three case reports we want to focus on some meaningful questions when treating patients with STS.

### 2.1. What Advantage Do We Have When Using PET/MRI in the Neoadjuvant Setting?

There is some evidence for the use of perioperative CTX, especially in younger patients. Several studies have shown conflicting results about adjuvant CTX in STS. Whereas some were in favor of CTX [20], other results were rather disappointing, because there was no benefit in overall survival [21, 22]. Meta-analysis found a significant advantage in relapse-free and overall survival [23, 24]. So it is still a matter of debate, which patients might have an advantage using adjuvant CTX [25, 26].

Therefore, the treatment guidelines of sarcoma research and treatment organizations have recommended a shared decision-making process considering among others age, tumour-subtype, grading, and size [27]. According to a retrospective French analysis only patients with grade 3 tumours benefit from adjuvant CTX [28].

 Nonetheless, there are still some problems and uncertainties about the neoadjuvant therapy. There is a substantial risk of progression with a potential risk of losing the chance of complete resection. Also, there are difficulties in the interpretation of response. RECIST criteria often do not match with clinical and histological responses to therapy. Cell death cannot be detected by conventional MRI diameter. Response in sarcoma can also occur with cystic changes that can mimic progressive disease.

**Figure 1:**
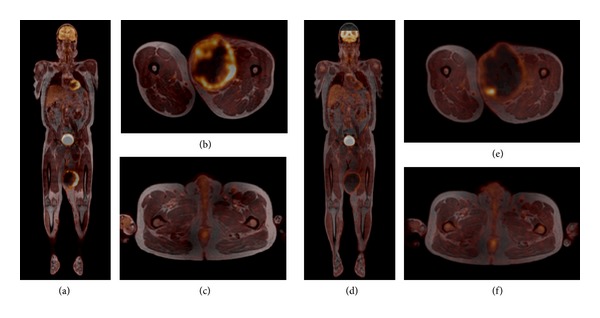
A 51-year-old male patient with rhabdomyosarcoma of the left thigh. Fused FDG PET/MR images before ((a)–(c)) and after chemotherapy ((d)–(f)). While the tumour size did not change significantly during chemotherapy ((d) and (e)), the FDG uptake is markedly reduced in comparison to the initial images ((a) and (b)). A light increase of FDG uptake is seen in some left inguinal lymph nodes; it did not change under chemotherapy ((c) and (f)), and the lymph nodes were proven to be benign by histology.

*Case 1*. A 51-year-old man presented with a rhabdomyosarcoma of the proximal thigh. The tumour measured 13 × 18 × 21 cm by CT scan with suspected metastases in the lung, lymph nodes, and bone. In order to improve knowledge about the actual tumour stage and plan possible multimodal treatment, functional imaging was additionally performed. PET/MRI showed a markedly elevated SUV uptake in the primary tumour (SUVmax = 7,6) and less activity in the inguinal lymph nodes (SUVmax = 2,5) (Figure 1). After two cycles of neoadjuvant therapy with epirubicin and ifosfamide the tumour showed a persisting FDG activity in SUVmax = 8.3, but a markedly decrease of cumulative SUVmean. At the same time, the tumour was progressive as measured by conventional criteria. The regional lymph nodes showed nonsignificant persistence of SUVmax.

It was decided to cancel further neoadjuvant treatment due to progression in size and move forward to resection. In the pathological report the tumour had 40% necrosis. Regional lymph nodes, which had been attributed to metastatic disease, were not involved. So far, there are no data of functional activity of the primary tumour compared to metastases in the same patient. Due to the discrepancy in activity of 4,0 in our patient, FDG-PET could help to distinguish between reactive and metastatic lymph nodes. Also, addition of a functional component could help to early detect responders in patients receiving neoadjuvant therapy. In this case, considering PET criteria could have given evidence to continue neoadjuvant systemic therapy. As outlined earlier, FDG-PET can give additional meaningful information for the decision-making process, but by today it cannot be considered the standard of care.

### 2.2. Is There a Need for PET/MR Imaging in Metastatic/Advanced Disease?

The main goal in palliative treatment of patients with STS is to reduce symptom burden and prolong progression-free or even overall survival. In light of emerging therapeutic options, it is important not to stick to futile therapeutic regimens.

**Figure 2:**
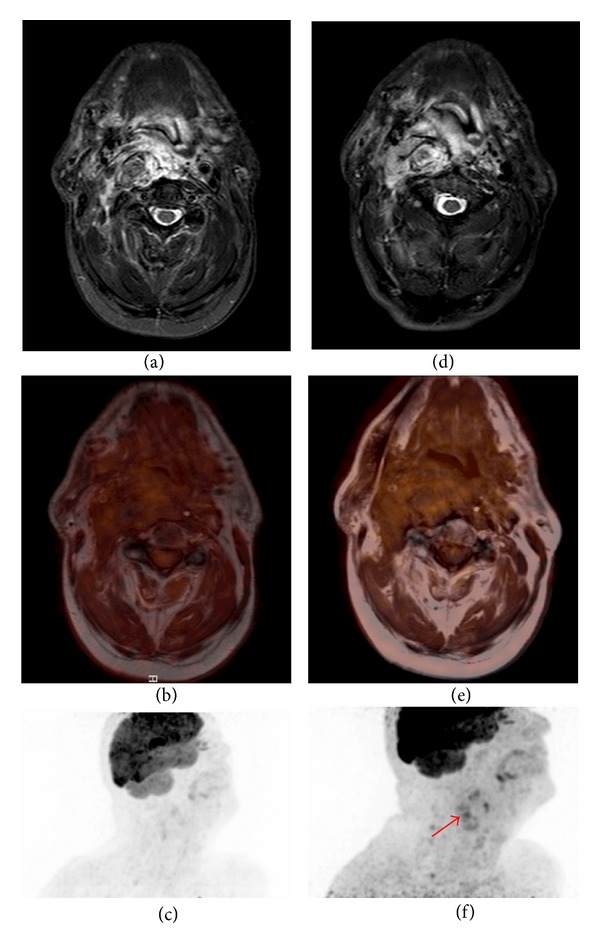
A 68-year-old patient with liposarcoma of the oropharynx. A maximum intensity projection (MIP) of the PET shows increased FDG uptake in the tumour ((f), arrow) as compared to the PET six months before (c). The short tau inversion recovery (STIR) images ((a) and (d)) and the corresponding fused PET/MR images ((d) and (e)) show no change in tumour size over six months.

*Case 2*. A 68-year-old patient presented with a recurrent, rapidly growing and inoperable G3 liposarcoma of the oropharynx. Primary resection and adjuvant radiotherapy had been completed one year before. After the initial CTX with epirubicine and ifosfamide the tumour remained stable (9 × 5 × 3 cm). The clinical problem was that a progressive tumour would rapidly cause a blocking of the airways. We therefore decided to use combined PET/MRI in addition to conventional imaging for early detection of tumour activity. After 6 months, the second followup showed a 67% increase in metabolic activity with no change in size at that time (Figure 2). We started 2nd-line systemic therapy with trabectedin and could detect tumour shrinkage after the first three cycles. So in this case, by adding the functional component the patient could be withheld from a dramatic complication to progressive disease without exerting additional toxicity.

### 2.3. Can PET/MR Resolve Discrepancies between Histological Grading and the Clinical Course of the Disease?

*Case 3*. In a 68-year-old female patient, after previous gross resection and long-term stabilization of an abdominal/retroperitoneal highly differentiated liposarcoma MRI showed a massive progression within only three months with features like necrosis often seen in high-grade sarcomas. This was accompanied by pollakisuria due to compression of the bladder and severe abdominal pain. Transformation to a high-grade liposarcoma was suspected. Further biopsies would be at risk of not being representative for a tumour harbouring regions with a potentially different grading.

**Figure 3:**
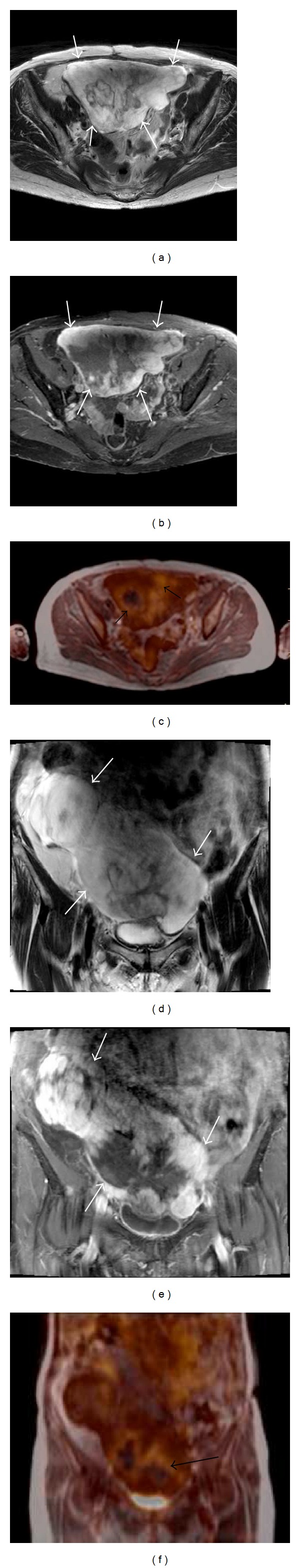
A 68-year-old patient with abdominal liposarcoma. The large tumour (white arrows) is well recognizable on both the T2-weighted images ((a) and (d)) and the contrast-enhanced, fat-saturated T1-weighted images ((b) and (e)). The fused PET/MR images ((c) and (f)) show an inhomogeneous FDG uptake (black arrows).

The tumour was 23 × 19 × 10 cm in size, partly necrotic, and showed only a slight uptake of FDG (Figure 3). Therefore, it was decided to resect the tumour. In case of a high-grade relapse we certainly would have favoured a systemic treatment approach. The histological examination confirmed the suspected relapse of the well-differentiated liposarcoma.

## 3. Conclusion

The new combined PET/MRI technique offers some interesting and promising diagnostic and therapeutic information to all clinicians, which have to deal with a magnitude of individual scenarios of patients with soft tissue sarcoma. We report on one case, where PET/MRI was used to guide neoadjuvant treatment. This is even more important, since it has been formerly demonstrated that in neoadjuvant treatment of soft tissue sarcoma PET is superior to conventional imaging in predicting pathological response [15]. Taking into account the potential prognostic value of preoperative PET [10], confirmatory studies on the value of perioperative chemotherapy in patients with high-risk soft tissue sarcomas are urgently needed. 

Furthermore, we report on a case with metastatic disease, where PET/MRI was able to contribute to the decision-making about the appropriate time for reinitiating chemotherapy. In clinical routine sometimes it might be of great value for the patient to anticipate the onset of progressive disease. Moreover, morphologic changes as part of response but mimicking progression defined by RECIST criteria may be misleading and prevent the patient from having clinical benefit by withdrawing an active drug.

The third case demonstrates that in large and heterogeneous tumours PET/MRI can help to guide tumour biopsy or judge the grading and thereby assist the clinician when considering different therapeutic options [8, 29]. So altogether, the combined modality of simultaneous PET/MRI offers new pieces to the puzzle of sarcoma treatment.
